# Modulation of the Liver–Brain Axis by Ondansetron in Thio-Acetamide-Induced Acute Hepatic Encephalopathy

**DOI:** 10.3390/ijms27209265

**Published:** 2026-10-19

**Authors:** Fatih Mehmet Sari, Yasin Bilgin, Betul Cicek, Engin Kurt, Mustafa Ozkaraca, Ali Gungor, Nezahat Kurt, Asli Ozbek Bilgin

**Affiliations:** 1 Department of Emergency Medicine, Faculty of Medicine, Erzincan Binali Yıldırım University, Erzincan 24100, Türkiye; drfmsari@gmail.com (F.M.S.); dr.ysn.blgn@gmail.com (Y.B.); 2 Department of Physiology, Faculty of Medicine, Erzincan Binali Yıldırım University, Erzincan 24100, Türkiye; betulll.cicekk@gmail.com; 3 Department of Emergency Medicine, Mengücek Gazi Training and Research Hospital, Erzincan 24100, Türkiye; engonge56@gmail.com; 4 Department of Pathology, Faculty of Veterinary Medicine, Cumhuriyet University, Sivas 58140, Türkiye; mustafaozkaraca@cumhuriyet.edu.tr; 5 Laboratory Technician and Veterinary Health Program, Health Services Vocational School, Osmaniye Korkut Ata University, Osmaniye 80000, Türkiye; aligungor@osmaniye.edu.tr; 6 Department of Biochemistry, Faculty of Medicine, Erzincan Binali Yıldırım University, Erzincan 24100, Türkiye; nezahat.kurt@erzincan.edu.tr; 7 R&D Activities and Research Laboratories, Faculty of Medicine, Erzincan Binali Yıldırım University, Erzincan 24100, Türkiye; 8 Department of Pharmacology, Faculty of Medicine, Erzincan Binali Yıldırım University, Erzincan 24100, Türkiye

**Keywords:** hepatic encephalopathy, ondansetron, liver-brain axis, neuroinflammation, oxidative stress, glutamine metabolism

## Abstract

Hepatic encephalopathy (HE) is a severe neurological complication of liver failure characterized by hyperammonemia, oxidative stress, neuroinflammation, and progressive neuronal dysfunction. Ondansetron (OND), a selective 5-hydroxytryptamine-3 (5-HT_3_) receptor antagonist, has reported anti-inflammatory and neuroactive properties, but its effects in HE remain poorly defined. This study evaluated OND in a thioacetamide (TAA)-induced acute HE model in female Sprague-Dawley rats. Animals were assigned to Control, TAA, OND, TAA+OND (0.5 mg/kg), and TAA+OND (1 mg/kg) groups. Serum and cerebral glutamine, oxidative and antioxidant parameters, histopathological alterations, and TLR2, NF-κB, IL-1β, GFAP, NLRP3, and caspase-1 immunoreactivity were assessed in liver and brain tissues. TAA increased glutamine accumulation, produced tissue injury, altered redox parameters, and increased several inflammation-related markers. OND significantly reduced cerebral glutamine and attenuated selected biochemical, histopathological, and immunohistochemical alterations, with generally more pronounced effects at 0.5 mg/kg. These findings indicate that OND administration is associated with partial modulation of metabolic, oxidative, inflammatory, and structural alterations in TAA-treated rats, warranting further receptor-specific and functional studies to clarify its mechanisms and therapeutic relevance.

## 1. Introduction

Hepatic encephalopathy (HE) is described as a dynamic problem in emergency medicine due to being one of the most frequent reasons for emergency department presentations in patients with liver cirrhosis and its high hospitalization and critical care unit rates [1,2]. HE is a brain disorder associated with acute and chronic liver disease. The pathophysiology differs from other neurological diseases, particularly due to hyperammonemia, altered blood-brain barrier function, oxidative stress, and neuroinflammation. In liver failure, impaired hepatic detoxification and urea synthesis lead to systemic ammonia accumulation. Ammonia entering the brain is predominantly detoxified in astrocytes through glutamine synthetase (GS)-mediated conversion of glutamate and ammonia to glutamine (GLN), resulting in intracellular glutamine accumulation that contributes to astrocytic swelling and cerebral dysfunction [3]. Excess ammonia and other neurotoxic metabolites reach the brain, causing astrocytic swelling, cerebral edema, altered neuronal integrity, abnormal neurotransmission, and inflammation that disrupt normal brain function and trigger behavioral changes, including motor, psychiatric, and neurocognitive alterations, which can progress to coma [4].

Astrocytic swelling is a major contributor to cerebral edema in acute HE and is driven by multiple interacting mechanisms, including hyperammonemia, oxidative/nitrosative stress, and neuroinflammation [3]. In HE, hyperammonemia is also known to trigger mitochondrial oxidative stress within microglial cells. This oxidative imbalance is characterized by the depletion of essential antioxidant defense systems, including superoxide dismutase (SOD), and catalase (CAT), alongside a significant elevation in malondialdehyde (MDA), a hallmark of lipid peroxidation [5]. Concurrently, the dysregulation of nitric oxide (NO) levels plays a dual role; while it contributes to cerebral vasodilation and subsequent edema, its interaction with reactive oxygen species leads to the formation of peroxynitrite, further exacerbating nitrosative stress and protein nitration within the brain parenchyma. This oxidative and nitrosative burden is coupled with increased myeloperoxidase (MPO) activity, an indicator of neutrophil infiltration [5]. Oxidative stress and damage-associated signals can promote activation of innate immune pathways, including Toll-like receptor (TLR) signaling [6]. Among these receptors, TLR2 can promote activation of nuclear factor-kappa B (NF-κB), a major transcriptional regulator of the inflammatory response, thereby promoting inflammatory gene transcription. Once activated, NF-κB not only upregulates the expression of various pro-inflammatory genes but also provides the necessary priming signal for the assembly of the nucleotide-binding oligomerization domain-like receptor protein 3 (NLRP3) inflammasome complex, an intracellular danger sensor. Following assembly of the NLRP3 inflammasome, pro-caspase-1 is cleaved to active caspase-1, which promotes the maturation of pro-IL-1β to biologically active IL-1β [7,8]. The release of IL-1β drives widespread neuroinflammation and blood-brain barrier dysfunction, playing a central role in the progression of the neurological impairments seen in HE [7]. This cascade further exacerbates neuroinflammation and reactive astrogliosis, as evidenced by increased GFAP expression, ultimately contributing to cerebral edema and neuronal dysfunction [8,9].

HE management remains challenging because current treatments primarily target ammonia reduction rather than the underlying neuroinflammatory and oxidative cascades. While liver transplantation is the only definitive treatment, its clinical use is limited due to high costs and graft scarcity, necessitating the search for novel molecules that target neuroinflammation [1]. In this study, the Thioacetamide (TAA) model was selected because it reproduces several hepatic and cerebral pathological features associated with acute HE. Reactive intermediates derived from TAA are known to impair mitochondrial function and cell permeability by causing extensive oxidative damage to proteins and lipids, and providing a pathophysiological environment representative of clinical HE [10].

Alterations in serotonergic neurotransmission have also been implicated in the pathophysiology of HE. Earlier studies of TAA-induced acute liver failure demonstrated increased indices of cerebral serotonergic activity and provided pharmacological evidence for the involvement of serotonergic mechanisms in experimental HE [11,12,13]. Subsequent reviews have further supported the presence of altered serotonin turnover and serotonergic neurotransmission in HE, although the direction and magnitude of these changes appear to depend on the experimental model, disease stage, and brain region [14,15]. More recently, Yang et al. demonstrated increased cortical 5-HT levels in experimental HE and linked this alteration to ammonia-dependent impairment of cerebral serotonin metabolism [16]. Collectively, these findings provide a rationale for investigating serotonergic modulation in HE; however, they do not establish a specific pathogenic role or activation of the 5-HT_3_ receptor.

Ondansetron (OND), a selective 5-hydroxytryptamine type 3 (5-HT_3_) receptor antagonist, has shown anti-inflammatory and neuroactive effects in several experimental settings [17,18,19]. Pharmacokinetic studies indicate that systemically administered ondansetron can reach the central nervous system, although its CNS penetration is limited. Ondansetron has been detected in cerebrospinal fluid following both oral and intravenous administration [20,21]. This pharmacological profile, together with evidence of altered serotonergic neurotransmission in HE, provided the rationale for investigating OND in the present model. Nevertheless, whether the observed effects of OND are mediated through direct 5-HT_3_ receptor-dependent mechanisms remains to be established. Therefore, the present study aimed to evaluate the effects of OND on metabolic, oxidative, inflammatory, and histopathological alterations in the liver and brain in a TAA-induced acute HE model.

## 2. Results

TAA administration produced hepatic and cerebral alterations consistent with the established acute HE model, including increased cerebral glutamine and marked histopathological injury in both liver and brain tissues.

### 2.1. Effects of OND on Serum and Cerebral Glutamine Levels

Serum glutamine levels differed significantly among the experimental groups (*p* < 0.001). Pairwise comparisons revealed significantly higher serum glutamine levels in the TAA group than in the control group (*p* = 0.009). Although OND treatment reduced serum glutamine levels compared with the TAA group, these reductions were not statistically significant after Bonferroni correction.

**Figure 1:**
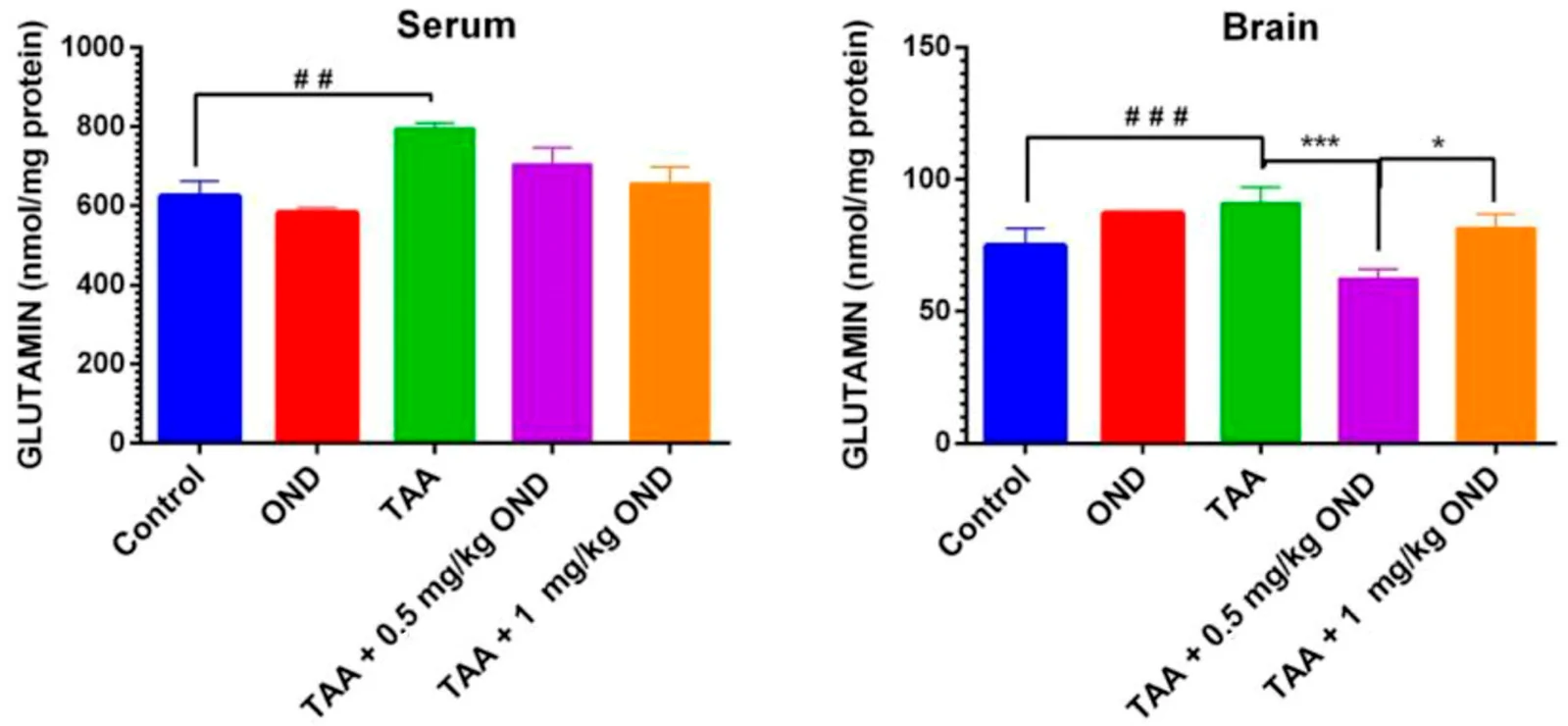
Effects of ondansetron (OND) on serum and brain glutamine levels in rats with thioacetamide (TAA)-induced hepatic encephalopathy. Data are presented as mean ± SD (*n* = 6 per group). Serum glutamine data were analyzed using the Kruskal-Wallis test followed by Bonferroni-adjusted pairwise comparisons. Cerebral glutamine data were analyzed using one-way ANOVA followed by Tukey’s multiple comparison test. ## means *p* < 0.01 and ### means *p* < 0.001 versus the control group; * for *p* < 0.05 and *** for *p* < 0.001 versus the TAA group.

Brain glutamine levels also differed significantly among the groups (*p* < 0.001). The TAA group exhibited significantly higher cerebral glutamine levels than the control group (*p* < 0.001). Treatment with OND at both 0.5 mg/kg (*p* < 0.001) and 1 mg/kg (*p* = 0.021) significantly reduced cerebral glutamine levels compared with the TAA group. No significant difference was observed between the TAA+OND (1 mg/kg) and control groups (Figure 1).

### 2.2. Effects of OND on Oxidative Stress Markers

**Figure 2:**
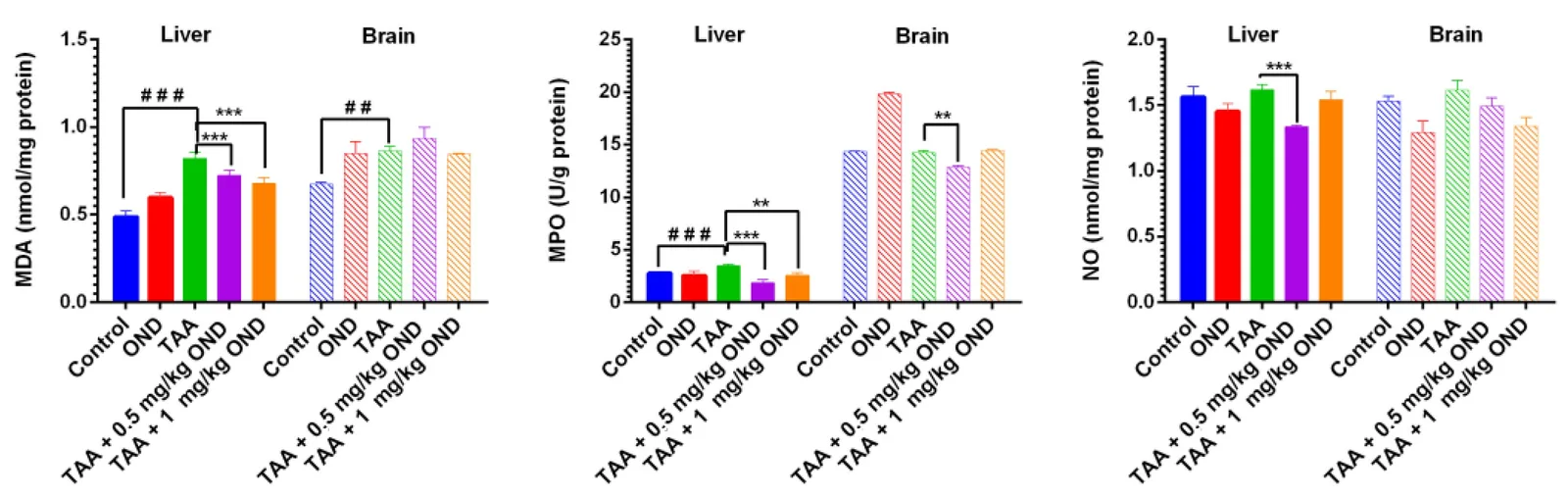
Effects of ondansetron (OND) on hepatic and cerebral oxidative stress markers in rats with thioacetamide (TAA)-induced hepatic encephalopathy. Malondialdehyde (MDA), myeloperoxidase (MPO), and nitric oxide (NO) levels were determined in liver and brain tissues. Data are presented as mean ± SD (*n* = 6 per group). Hepatic MDA data were analyzed using one-way ANOVA followed by Tukey’s multiple comparison test. Hepatic MPO, and NO data were analyzed using Welch’s ANOVA followed by Games-Howell post hoc test. Cerebral MDA and MPO, data were analyzed using one-way ANOVA followed by Games-Howell post hoc test. Cerebral NO data were analyzed using one-way ANOVA followed by Tukey’s multiple comparison test. ## *p* < 0.01 and ### *p* < 0.001 versus the control group; ** *p* < 0.01 and *** *p* < 0.001 versus the TAA group. Abbreviations: MDA, malondialdehyde; MPO, myeloperoxidase; NO, nitric oxide.

Hepatic MDA levels differed significantly among the experimental groups. The TAA group exhibited significantly higher hepatic MDA levels than the control group (*p* < 0.001). Treatment with OND at both 0.5 and 1 mg/kg significantly (both ** *p* < 0.001) reduced hepatic MDA levels compared with the TAA group, although values remained significantly higher than those of the control group. No significant difference was observed between the two OND treatment groups. In the brain, MDA levels also differed significantly among the groups (Figure 2). Cerebral MDA levels were significantly higher in the TAA group than in the control group (*p* < 0.01). However, neither OND treatment significantly altered cerebral MDA levels compared with the TAA group.

Hepatic MPO activity was significantly increased in the TAA group compared with the control group (*p* < 0.001). Treatment with OND significantly reduced hepatic MPO activity, with a more pronounced reduction observed in the TAA+OND (0.5 mg/kg) group (*p* < 0.001 vs. TAA) than in the TAA+OND (1 mg/kg) group (*p* < 0.01 vs. TAA). In the brain, MPO activity was highest in the OND-alone group. Compared with the TAA group, cerebral MPO activity was significantly reduced only in the TAA+OND (0.5 mg/kg) group (*p* < 0.01), whereas no significant difference was observed between the TAA and TAA+OND (1 mg/kg) groups.

Hepatic NO levels also differed among the experimental groups. The TAA+OND (0.5 mg/kg) group exhibited significantly lower hepatic NO levels than the TAA group (*p* < 0.001), whereas no significant difference was observed between the TAA and TAA+OND (1 mg/kg) groups. In the brain, NO levels showed no significant differences between the TAA group and either OND-treated group.

### 2.3. Effects of OND on Antioxidant Defense Markers

**Figure 3:**
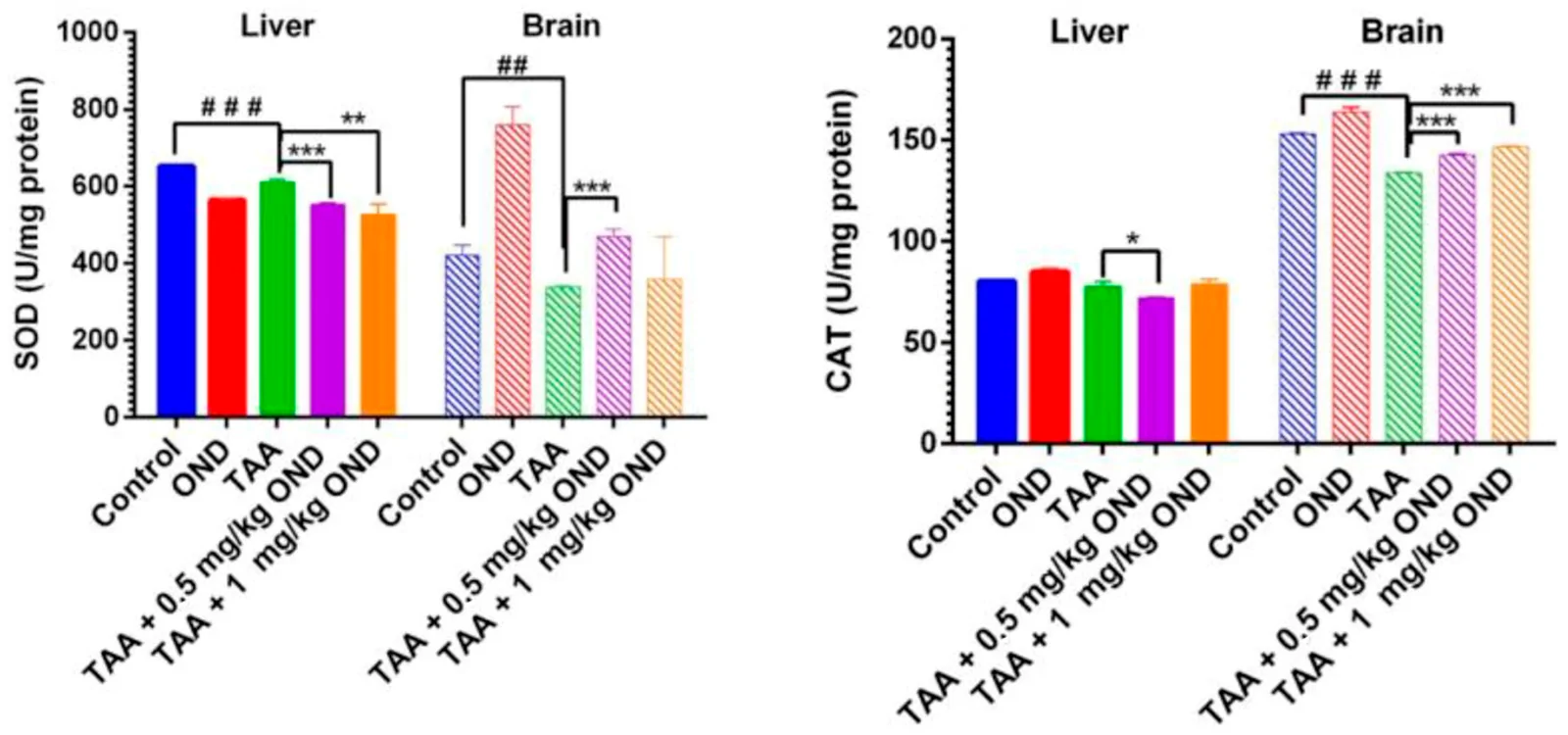
Effects of ondansetron (OND) on hepatic and cerebral antioxidant defense markers in rats with thioacetamide (TAA)-induced hepatic encephalopathy. Superoxide dismutase (SOD) activity and catalase (CAT) activity were determined in liver and brain tissues. Data are presented as mean ± SD (*n* = 6 per group). Hepatic SOD, hepatic CAT, and cerebral CAT data were analyzed using Welch’s ANOVA followed by the Games-Howell post hoc test. Cerebral SOD data were analyzed using one-way ANOVA followed by Tukey’s multiple comparison test. ## means *p* < 0.01 and ### means *p* < 0.001 versus the control group; * means *p* < 0.05, ** means *p* < 0.01, and *** means *p* < 0.001 versus the TAA group. Abbreviations: SOD, superoxide dismutase; CAT, catalase.

Hepatic SOD activity differed significantly among the experimental groups. The TAA group exhibited significantly lower hepatic SOD activity than the control group (*p* < 0.001). Treatment with OND significantly increased hepatic SOD activity compared with the TAA group, with a more pronounced increase observed in the TAA+OND (0.5 mg/kg) group (*p* < 0.001 vs. TAA) than in the TAA+OND (1 mg/kg) group (*p* < 0.01 vs. TAA). In the brain, SOD activity was significantly reduced in the TAA group compared with the OND-alone group (*p* < 0.01). Among the treatment groups, only TAA+OND (0.5 mg/kg) significantly increased cerebral SOD activity compared with the TAA group (*p* < 0.001), whereas no significant difference was observed between the TAA and TAA+OND (1 mg/kg) groups (Figure 3).

The TAA+OND (0.5 mg/kg) group showed significantly lower hepatic CAT activity than the TAA group (*p* < 0.05), whereas no significant difference was observed between the TAA and TAA+OND (1 mg/kg) groups. In the brain, CAT activity was significantly decreased in the TAA group compared with the control group (*p* < 0.001). Treatment with OND at both 0.5 and 1 mg/kg significantly increased cerebral CAT activity compared with the TAA group (both ** *p* < 0.001).

### 2.4. Effects of OND Treatment on Histopathological Alterations in TAA-Induced HE in Rats

#### 2.4.1. Liver Histopathology

**Figure 4:**
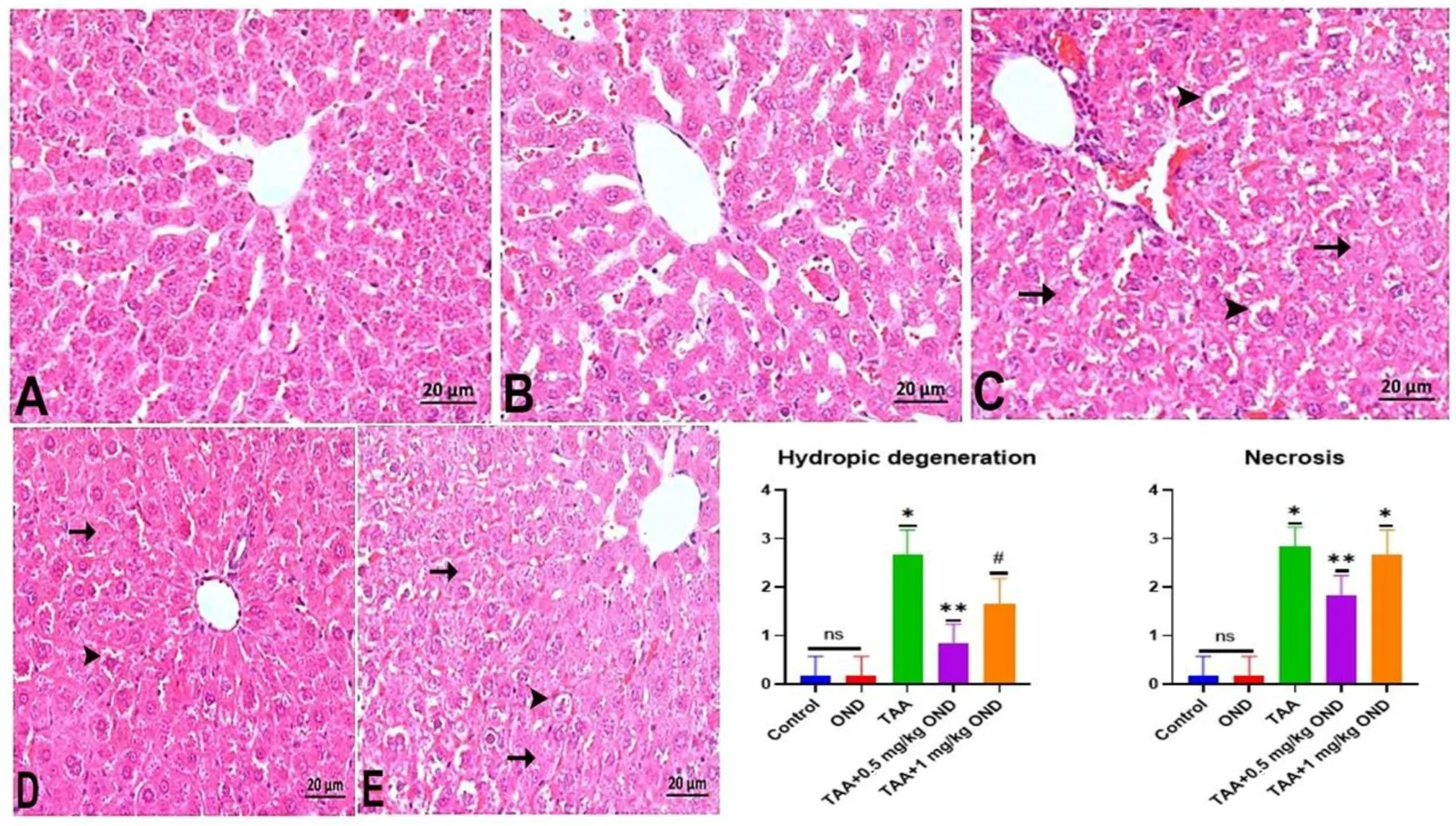
Effects of ondansetron (OND) on TAA-induced hepatic histopathological alterations. Representative hematoxylin and eosin (H&E)-stained liver sections and corresponding semiquantitative scores for hydropic degeneration and hepatocellular necrosis are shown. (**A**) Control group showing normal hepatic architecture; (**B**) OND-alone group showing normal histological appearance; (**C**) TAA group showing severe hydropic degeneration (arrowheads) and hepatocellular necrosis (arrows); (**D**) TAA+OND (0.5 mg/kg) group showing mild hydropic degeneration and moderate hepatocellular necrosis; and (**E**) TAA+OND (1 mg/kg) group showing moderate hydropic degeneration and severe hepatocellular necrosis. Scale bar = 20 μm. Histopathological alterations were scored semiquantitatively as absent (0), mild (1), moderate (2), or severe (3). Data are presented as mean ± SD (*n* = 6 per group). Group differences were evaluated using the Kruskal-Wallis test. Symbols (*, **, #) indicate statistically significant differences between groups (*p* < 0.05); ns, not significant.

Semiquantitative histopathological evaluation revealed significant differences in hepatic hydropic degeneration and necrosis among the experimental groups (Figure 4). No remarkable histopathological alterations were observed in the control or OND-alone groups. TAA administration resulted in severe hydropic degeneration and hepatocellular necrosis. OND treatment reduced the severity of hydropic degeneration, with mild changes in the TAA+OND (0.5 mg/kg) group and moderate changes in the TAA+OND (1 mg/kg) group. Hepatocellular necrosis was reduced to a moderate degree in the TAA+OND (0.5 mg/kg) group, whereas severe necrotic changes remained evident in the TAA+OND (1 mg/kg) group (Figure 4).

#### 2.4.2. Brain Histopathology

**Figure 5:**
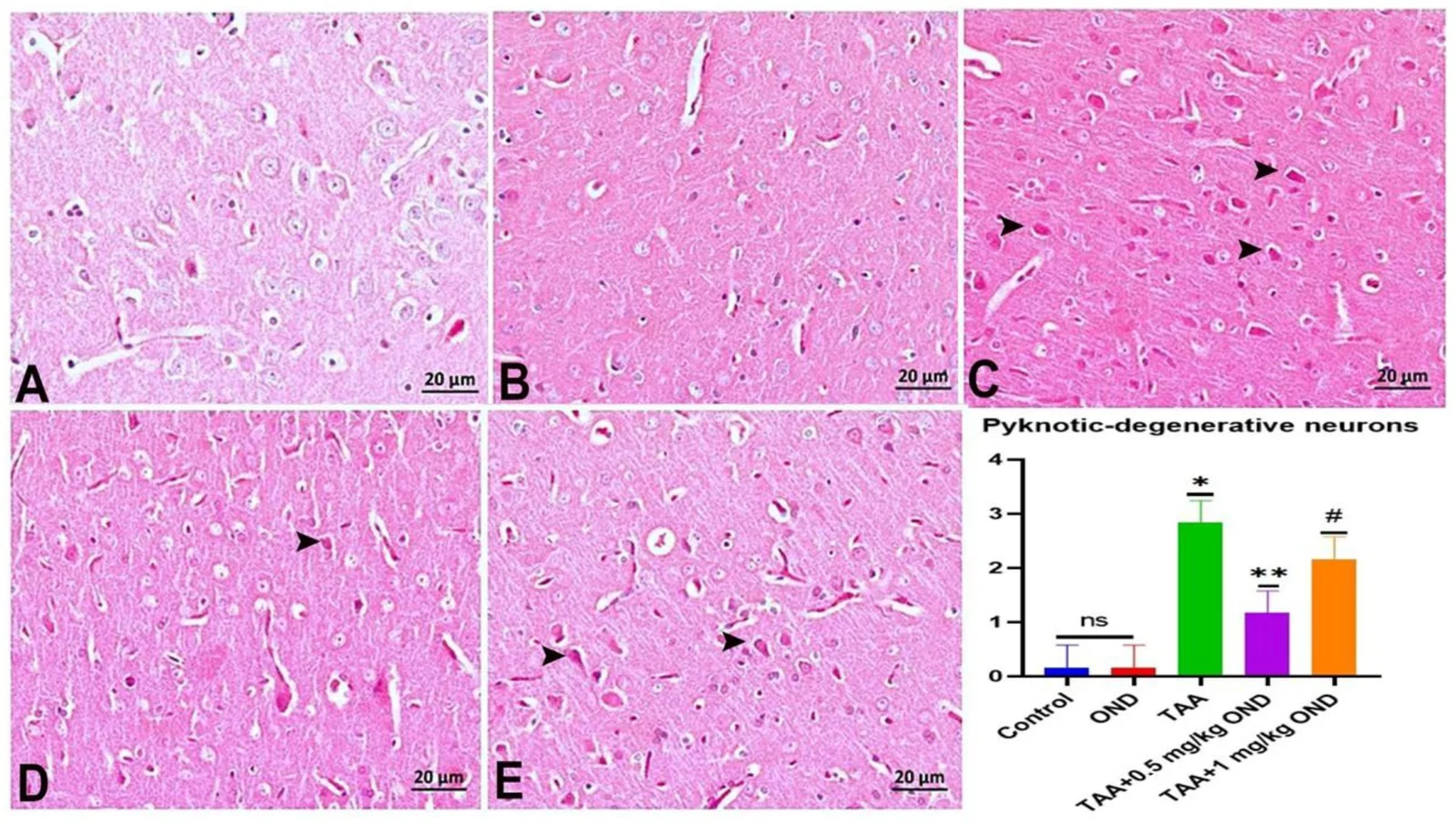
Effects of ondansetron (OND) on TAA-induced cerebral histopathological alterations. Representative hematoxylin and eosin (H&E)-stained brain sections and corresponding semiquantitative scores for pyknotic-degenerative neuronal changes are shown. (**A**) Control group showing normal histological architecture; (**B**) OND-alone group showing normal histological appearance; (**C**) TAA group showing severe pyknotic-degenerative neuronal changes (arrowheads); (**D**) TAA+OND (0.5 mg/kg) group showing mild pyknotic-degenerative neuronal changes; and (**E**) TAA+OND (1 mg/kg) group showing moderate pyknotic-degenerative neuronal changes. Scale bar = 20 μm. Histopathological alterations were scored semiquantitatively as absent (0), mild (1), moderate (2), or severe (3). Data are presented as mean ± SD (*n* = 6 per group). Group differences were evaluated using the Kruskal-Wallis test. Symbols (*, **, #) indicate statistically significant differences between groups (*p* < 0.05); ns, not significant.

In the brain, the TAA group exhibited severe pyknotic and degenerative neuronal changes. OND treatment was associated with reduced histopathological severity, with mild pyknotic-degenerative changes observed in the TAA+OND (0.5 mg/kg) group and moderate changes in the TAA+OND (1 mg/kg) group (Figure 5).

### 2.5. Effects of OND on Immunohistochemical Findings in TAA-Induced HE in Rats

**Figure 6:**
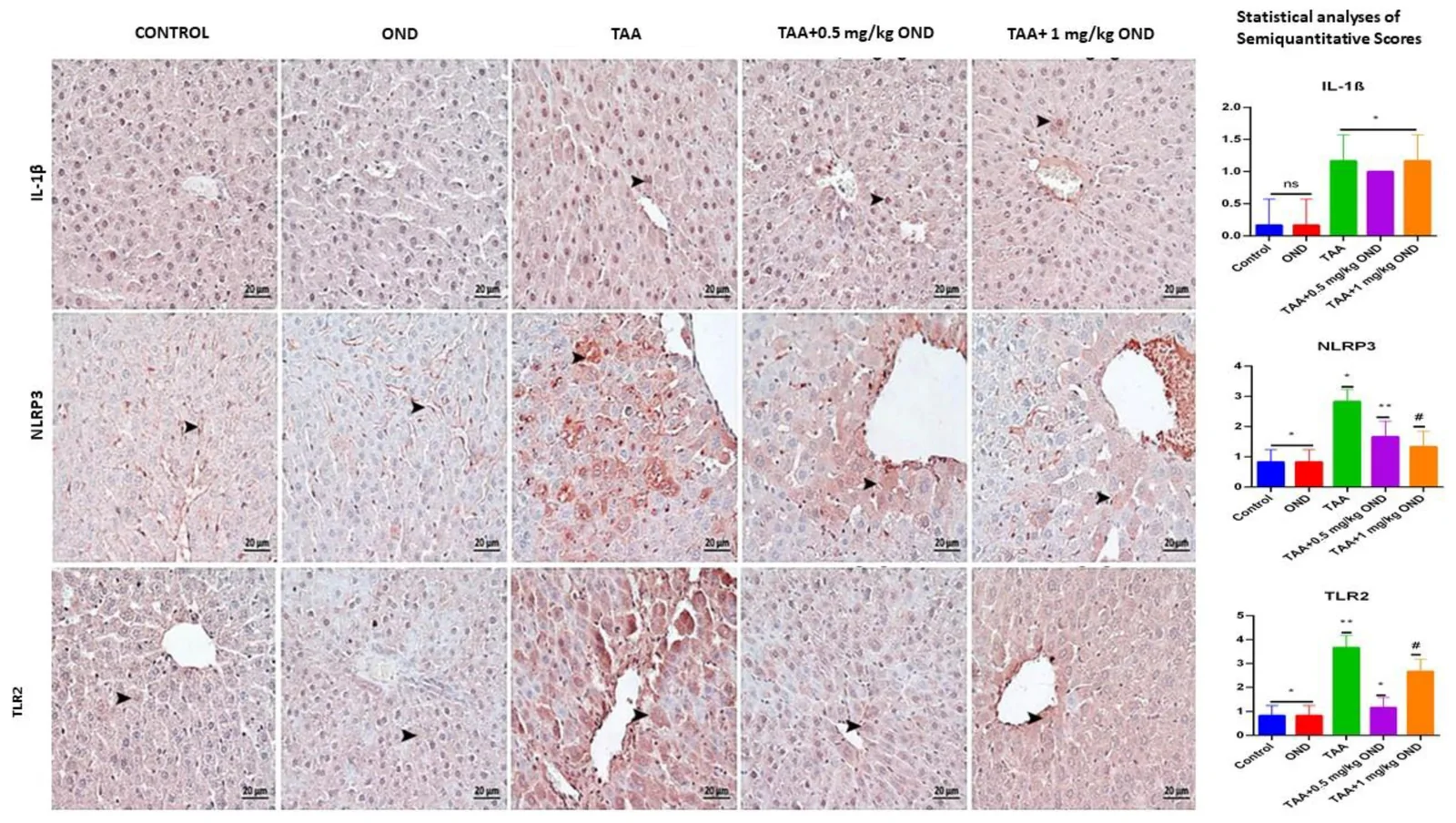
Effects of ondansetron (OND) on IL-1β, NLRP3, and TLR2 immunoreactivity in liver tissue from rats with TAA-induced acute hepatic encephalopathy. Representative immunohistochemical images and corresponding semiquantitative scores are shown. Columns represent the experimental groups: Control, OND, TAA, TAA+OND (0.5 mg/kg), and TAA+OND (1 mg/kg), whereas rows represent IL-1β, NLRP3, and TLR2, respectively. Arrowheads indicate immunopositive staining. Scale bar = 20 μm. Immunoreactivity was scored semiquantitatively as negative (0), weak (1), moderate (2), strong (3), or very strong (4). Data are presented as mean ± SD (*n* = 6 per group). Group differences were evaluated using the Kruskal-Wallis test. Symbols (*, **, #) indicate statistically significant differences between groups (*p* < 0.05); ns, not significant.

**Figure 7:**
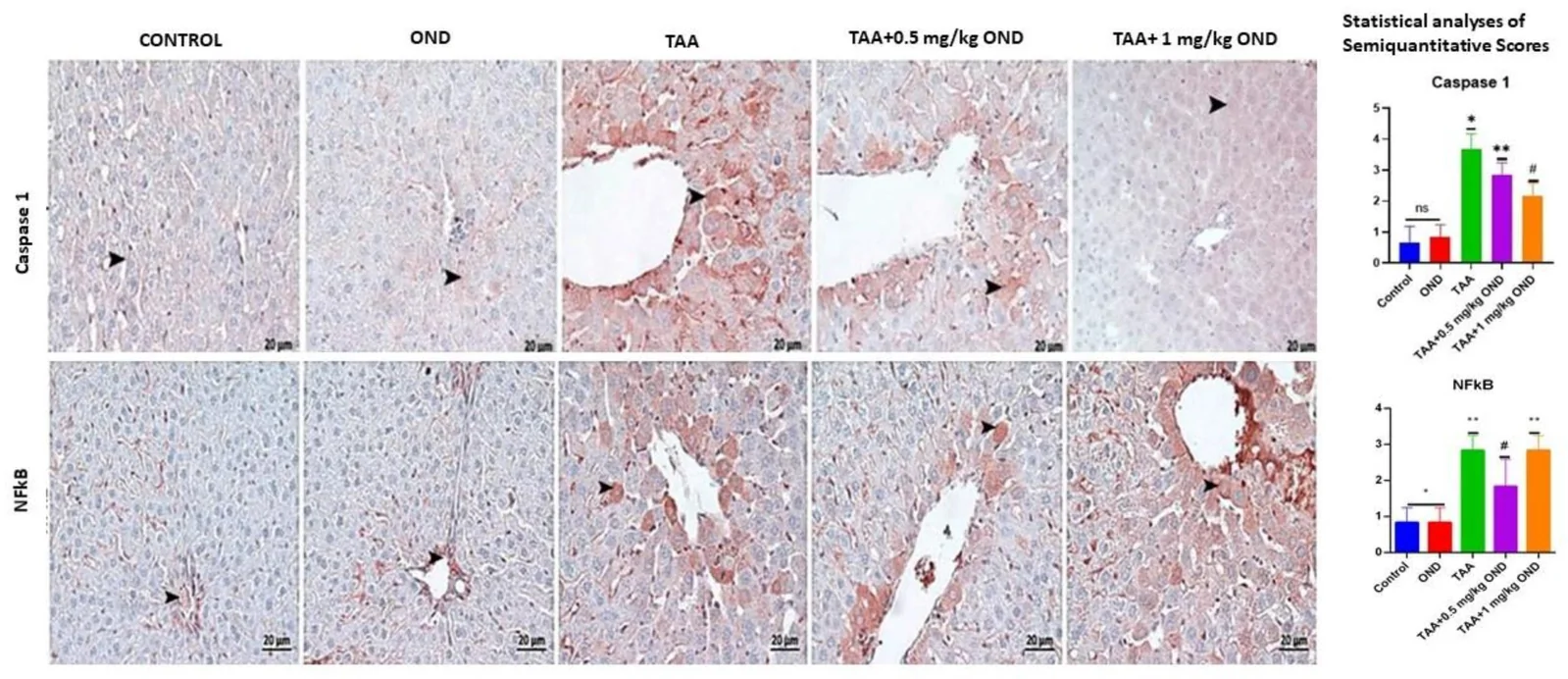
Effects of ondansetron (OND) on caspase-1 and NF-κB immunoreactivity in liver tissue from rats with TAA-induced acute hepatic encephalopathy. Representative immunohistochemical images and corresponding semiquantitative scores are shown. Columns represent the experimental groups: Control, OND, TAA, TAA+OND (0.5 mg/kg), and TAA+OND (1 mg/kg), whereas rows represent caspase-1 and NF-κB, respectively. Arrowheads indicate immunopositive staining. Scale bar = 20 μm. Immunoreactivity was scored semiquantitatively as negative (0), weak (1), moderate (2), strong (3), or very strong (4). The caspase-1 antibody detects total caspase-1 protein and does not specifically distinguish the cleaved/active form; therefore, caspase-1 staining represents total caspase-1 immunoreactivity rather than a direct measure of caspase-1 activation. Data are presented as mean ± SD (*n* = 6 per group). Group differences were evaluated using the Kruskal-Wallis test. Symbols (*, **, #) indicate statistically significant differences between groups (*p* < 0.05); ns, not significant.

**Figure 8:**
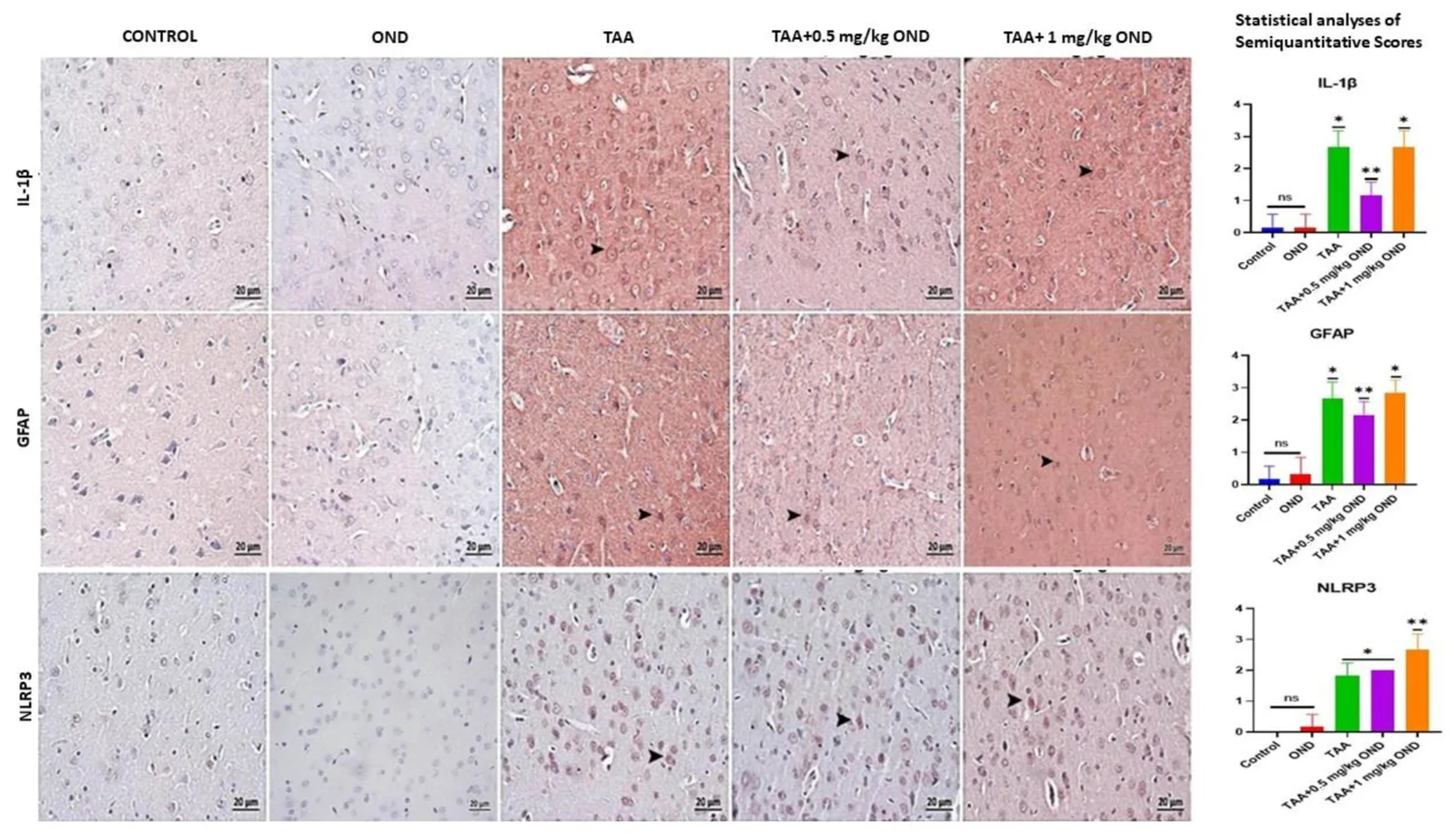
Effects of ondansetron (OND) on IL-1β, GFAP, and NLRP3 immunoreactivity in brain tissue from rats with TAA-induced acute hepatic encephalopathy. Representative immunohistochemical images and corresponding semiquantitative scores are shown. Columns represent the experimental groups: Control, OND, TAA, TAA+OND (0.5 mg/kg), and TAA+OND (1 mg/kg), whereas rows represent IL-1β, GFAP, and NLRP3, respectively. Arrowheads indicate immunopositive staining. Scale bar = 20 μm. Immunoreactivity was scored semiquantitatively as negative (0), weak (1), moderate (2), strong (3), or very strong (4). Data are presented as mean ± SD (*n* = 6 per group). Group differences were evaluated using the Kruskal-Wallis test. Symbols (*, **) indicate statistically significant differences between groups (*p* < 0.05); ns, not significant.

**Figure 9:**
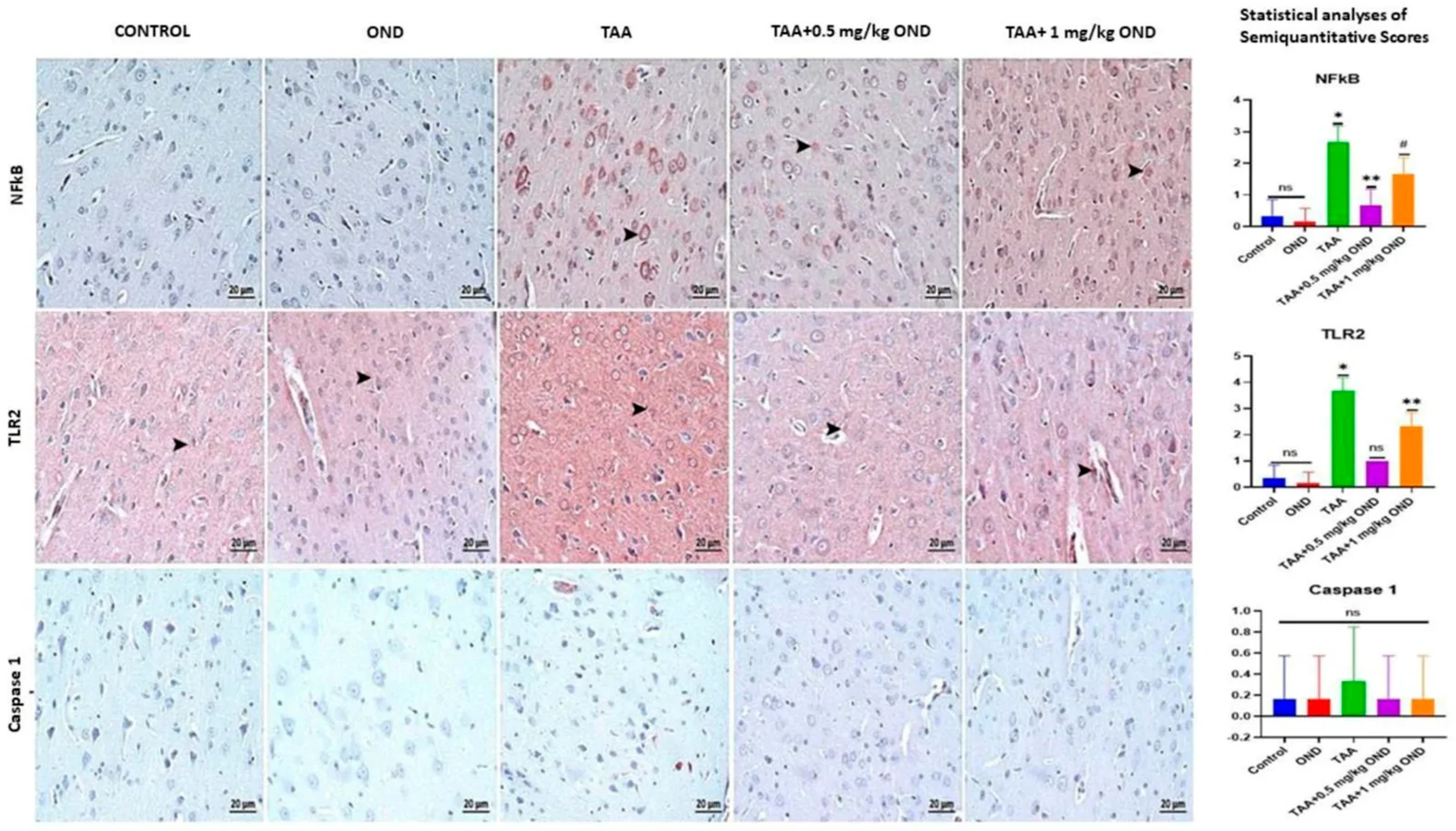
Effects of ondansetron (OND) on NF-κB, TLR2, and caspase-1 immunoreactivity in brain tissue from rats with TAA-induced acute hepatic encephalopathy. Representative immunohistochemical images and corresponding semiquantitative scores are shown. Columns represent the experimental groups: Control, OND, TAA, TAA+OND (0.5 mg/kg), and TAA+OND (1 mg/kg), whereas rows represent NF-κB, TLR2, and caspase-1, respectively. Arrowheads indicate immunopositive staining. Scale bar = 20 μm. Immunoreactivity was scored semiquantitatively as negative (0), weak (1), moderate (2), strong (3), or very strong (4). Data are presented as mean ± SD (*n* = 6 per group). Group differences were evaluated using the Kruskal-Wallis test. No significant differences in caspase-1 immunoreactivity were observed among the experimental groups. Symbols (*, **, #) indicate statistically significant differences between groups (*p* < 0.05); ns, not significant.

Immunohistochemical evaluation demonstrated significant differences in the immunoreactivity of several inflammation-related markers among the experimental groups in both liver and brain tissues. Representative immunohistochemical images and corresponding semiquantitative scores are presented together in Figure 6, Figure 7, Figure 8 and Figure 9.

#### 2.5.1. Liver Immunohistochemistry

In the liver, IL-1β immunoreactivity was absent in the control and OND-alone groups and weak in the TAA-treated groups. NLRP3 immunoreactivity was strong in the TAA group, moderate in the TAA+OND (0.5 mg/kg) group, and weak in the TAA+OND (1 mg/kg) group. TLR2 immunoreactivity was very strong in the TAA group, weak in the control, OND-alone, and TAA+OND (0.5 mg/kg) groups, and strong in the TAA+OND (1 mg/kg) group. Caspase-1 immunoreactivity was weak in the control and OND-alone groups, very strong in the TAA group, strong in the TAA+OND (0.5 mg/kg) group, and moderate in the TAA+OND (1 mg/kg) group. The caspase-1 antibody used in the present study detects total caspase-1 protein and does not specifically distinguish the cleaved/active form; therefore, the findings reported here represent total caspase-1 immunoreactivity rather than a direct measure of caspase-1 activation. NF-κB immunoreactivity was strong in the TAA and TAA+OND (1 mg/kg) groups and moderate in the TAA+OND (0.5 mg/kg) group (Figure 6 and Figure 7).

#### 2.5.2. Brain Immunohistochemistry

In the brain, no appreciable immunoreactivity for IL-1β, GFAP, NLRP3, NF-κB, or caspase-1 was observed in the control or OND-alone groups, whereas weak TLR2 immunoreactivity was detected. IL-1β and NF-κB immunoreactivity was strongest in the TAA group, weak in the TAA+OND (0.5 mg/kg) group, and moderate in the TAA+OND (1 mg/kg) group. GFAP immunoreactivity was strong in both the TAA and TAA+OND (1 mg/kg) groups and moderate in the TAA+OND (0.5 mg/kg) group. NLRP3 immunoreactivity was moderate in the TAA and TAA+OND (0.5 mg/kg) groups but strong in the TAA+OND (1 mg/kg) group. TLR2 immunoreactivity was very strong in the TAA group, weak in the control, OND-alone, and TAA+OND (0.5 mg/kg) groups, and moderate in the TAA+OND (1 mg/kg) group. Caspase-1 immunoreactivity was not detected in any experimental group (Figure 8 and Figure 9).

## 3. Discussion

The present study shows that ondansetron administration was associated with im-provements in several biochemical, histopathological, and neuroinflammatory alterations induced by TAA in liver and brain tissues. TAA administration disrupted glutamine homeostasis, increased selected oxidative stress markers, and induced histopathological damage accompanied by altered immunoreactivity of inflammatory markers, including TLR2, NF-κB, IL-1β, GFAP, and NLRP3. OND treatment ameliorated several of these alterations, with the 0.5 mg/kg dose generally producing more pronounced effects than the 1 mg/kg dose. These findings indicate that OND administration is associated with modulation of metabolic, oxidative, inflammatory, and structural changes within the liver-brain axis; however, the present study does not establish a specific receptor-dependent mechanism.

The pathophysiological and therapeutic context of HE differs between acute and chronic liver failure. In acute liver failure, abrupt hyperammonemia promotes astrocyte swelling and cerebral edema, whereas chronic HE develops in the setting of sustained liver dysfunction and/or portosystemic shunting, with contributions from altered ammonia metabolism, gut dysbiosis, and systemic inflammation. Emerging evidence also highlights the liver-spleen axis as an immune-metabolic network contributing to inflammation in chronic liver disease [22], although its specific role in HE remains to be established. Thus, acute HE requires particular attention to cerebral edema and neuroprotection, whereas chronic HE management focuses more on long-term control of ammonia production and systemic precipitating factors. Because the present TAA model represents acute toxic liver injury, our findings should primarily be interpreted within the context of acute HE rather than directly extrapolated to chronic HE.

The rationale for investigating OND in the present model is supported by evidence of altered serotonergic neurotransmission during HE. In TAA-induced acute liver failure, increased cerebral serotonergic activity and pharmacological modulation of HE-related behavioral manifestations by serotonin antagonists have been reported [11,12,13]. More recently, increased cortical 5-HT concentrations were linked to ammonia-dependent impairment of cerebral serotonin metabolism [16]. These findings support serotonergic dysregulation in experimental HE but do not demonstrate specific involvement of the 5-HT3 receptor. As a selective 5-HT3 receptor antagonist with established central pharmacological actions, OND was investigated on this pharmacological rationale; however, because 5-HT3 receptor expression was not assessed in the present model, the observed effects cannot be attributed specifically to 5-HT3 receptor-dependent signaling.

A central contribution to the observed effects is pharmacologically plausible because systemically administered OND exhibits limited but measurable CNS penetration. No specific formulation or BBB-targeting strategy was used in the present study. OND has been directly quantified in rat brain following systemic administration [23], and human pharmacokinetic studies have demonstrated measurable CSF exposure, although substantially lower than plasma exposure [20,21]. Nevertheless, brain OND concentrations and target engagement were not assessed in the present study. In addition, central 5-HT3 receptors exhibit heterogeneous regional and cellular distribution, including expression in the cerebral cortex, hippocampus, amygdala, olfactory bulb, and brainstem [24]. Because region-specific receptor expression, occupancy, and cell-specific target engagement were not evaluated, the observed cerebral effects cannot be assigned to a specific anatomical region or cell population or attributed specifically to central 5-HT3 receptor blockade.

One of the most consistent biochemical findings was the marked increase in glutamine following TAA administration. OND had a more pronounced effect on cerebral than circulating glutamine: serum glutamine showed only a non-significant tendency to decrease, whereas brain glutamine was significantly reduced in both OND-treated groups, with the greatest reduction at 0.5 mg/kg. This finding is consistent with the established role of the ammonia-glutamine pathway in HE, in which cerebral ammonia is detoxified by astrocytic glutamine synthetase, leading to glutamine accumulation [25,26]. Excessive intracellular glutamine may contribute to astrocytic swelling, mitochondrial dysfunction, oxidative/nitrosative stress, and neuronal injury [25]. However, cerebral glutamine cannot be considered a direct surrogate for systemic ammonia concentrations [26]. Because blood ammonia and glutamine synthetase activity were not measured, the reduction in cerebral glutamine cannot be interpreted as evidence of reduced systemic hyperammonemia or improved hepatic ammonia detoxification. The parallel changes in cerebral glutamine and GFAP immunoreactivity are nevertheless noteworthy and may reflect coordinated metabolic and astrocytic responses associated with OND treatment.

The reduction in cerebral glutamine was accompanied by changes in selected oxidative and antioxidant parameters. TAA administration produced tissue-specific alterations in redox-related markers, with more consistent changes observed in the liver than in the brain. In agreement with previous experimental studies, TAA-induced HE was associated with alterations in MDA, MPO, and NO levels, although the magnitude and statistical significance of these changes varied according to tissue and marker [5,10]. These alterations are consistent with the involvement of oxidative and nitrosative stress in HE pathophysiology. Excessive production of reactive oxygen and nitrogen species has been recognized as a major contributor to hepatocellular injury and secondary neurological damage through oxidation of membrane lipids, proteins, and nucleic acids, ultimately promoting neuronal dysfunction and neuroinflammation [7].

OND produced tissue- and marker-specific effects on redox-related parameters rather than uniform normalization of redox homeostasis. The effects were more consistent in the liver, where OND reduced MDA and MPO and, at 0.5 mg/kg, also lowered NO. Cerebral responses were more heterogeneous, as OND did not significantly alter cerebral MDA or NO, while cerebral MPO showed a distinct pattern, including the highest activity in the OND-alone group. This unexpected finding cannot be explained by the present data and warrants cautious interpretation. OND also partially improved SOD activity in TAA-treated animals, whereas changes in CAT were modest and tissue- and dose-dependent. Overall, these findings are consistent with partial modulation of redox homeostasis following OND treatment rather than uniform activation of antioxidant defenses [17,18,19].

The hepatic effects of OND should be interpreted cautiously because the present study does not establish direct 5-HT3 receptor-mediated actions in hepatocytes. The observed changes may instead involve indirect systemic, immune, vascular, neural, or redox-related mechanisms, which cannot be distinguished without receptor- and cell-specific analyses. In addition, the findings did not demonstrate a conventional linear dose-response relationship, as 0.5 mg/kg generally produced more pronounced effects than 1 mg/kg. Pharmacodynamic saturation and altered OND disposition secondary to TAA-induced hepatic dysfunction could theoretically contribute to this pattern; however, these explanations remain hypothetical because only two doses were evaluated and OND exposure and receptor occupancy were not assessed. Thus, 0.5 mg/kg should be regarded as the dose associated with the most pronounced effects within the tested range rather than as an established optimal dose.

An additional consideration in interpreting this non-linear dose-related pattern is the pharmacokinetic behavior of OND in hepatic dysfunction. OND is predominantly eliminated through hepatic metabolism, and hepatic impairment is associated with reduced clearance and increased systemic exposure [27,28]. This may be relevant to the present TAA model, in which substantial hepatic injury could alter OND disposition and potentially contribute to the observed non-linear dose-related pattern. However, plasma OND concentrations, metabolites, and hepatic drug-metabolizing enzyme activity were not assessed; therefore, altered OND metabolism remains a pharmacokinetic consideration rather than a demonstrated mechanism. Rare aminotransferase elevations have also been reported with OND [29,30], supporting the importance of hepatic safety assessment in patients with impaired liver function.

Histopathological findings provided complementary evidence of tissue-level injury. TAA induced extensive pyknotic neuronal changes in the brain and severe hydropic degeneration and hepatocellular necrosis in the liver, consistent with structural alterations previously reported in experimental HE [10,26]. OND attenuated these changes, particularly at 0.5 mg/kg, with fewer degenerative neurons and reduced hepatic hydropic degeneration and necrosis. These morphological findings paralleled several of the biochemical alterations observed following OND treatment.

Immunohistochemical analyses further demonstrated alterations in inflammatory markers associated with TAA-induced HE. TAA increased TLR2 and NF-κB immunoreactivity in both liver and brain tissues, whereas OND attenuated these changes, generally more prominently at 0.5 mg/kg. These reductions were accompanied by decreased IL-1β immunoreactivity. TLR-mediated signaling can promote NF-κB-dependent transcription of pro-inflammatory mediators, including IL-1β [6,8]. However, because immunohistochemistry assessed overall immunoreactivity rather than receptor activation or pathway activity, these findings demonstrate an association with reduced inflammatory signaling-related markers but do not establish functional inhibition of the TLR2/NF-κB pathway or its dependence on 5-HT3 receptor antagonism.

TAA also markedly increased GFAP immunoreactivity, consistent with enhanced astrocytic reactivity, whereas OND attenuated this response, particularly at 0.5 mg/kg. Astrocytes are central to cerebral ammonia detoxification and glutamine metabolism in HE [25,26], making the parallel changes in GFAP immunoreactivity and cerebral glutamine noteworthy. However, these findings do not establish a causal relationship between astrocytic reactivity and glutamine accumulation. Moreover, because cell-specific co-localization was not performed, the cellular sources of the accompanying inflammatory changes cannot be determined.

The inflammatory markers evaluated may also be considered within an interconnected inflammasome-related framework. TLR/NF-κB signaling contributes to the priming of NLRP3 and pro-IL-1β, whereas NLRP3 inflammasome assembly can promote caspase-1-dependent maturation of IL-1β. Experimental studies further support interactions among hyperammonemia, oxidative stress, NF-κB/NLRP3 signaling, and pro-inflammatory mediators in HE [7,31]. In the present study, TAA increased hepatic NLRP3 and caspase-1 immunoreactivity, both of which were reduced following OND treatment. In the brain, TAA increased NLRP3 immunoreactivity, whereas caspase-1 immunoreactivity did not differ significantly among groups. Importantly, the caspase-1 antibody detects total caspase-1 rather than specifically its cleaved/active form. Therefore, these semiquantitative immunohistochemical findings indicate alterations in inflammasome-related markers but do not establish functional NLRP3 inflammasome activation or inhibition. The lack of further reduction in cerebral NLRP3 immunoreactivity at 1 mg/kg was also consistent with the overall non-linear dose-related pattern observed across several endpoints in the present study.

Taken together, the present findings can be integrated within the established pathophysiological framework of acute HE, in which impaired hepatic ammonia handling promotes cerebral glutamine accumulation, astrocytic dysfunction, oxidative/nitrosative stress, neuroinflammation, and ultimately neuronal injury [25,26]. Consistent with different levels of this cascade, TAA increased cerebral glutamine and was associated with altered GFAP immunoreactivity, redox imbalance, inflammatory and inflammasome-related changes, and neuronal histopathological injury. OND attenuated several of these abnormalities, particularly at 0.5 mg/kg, providing evidence of modulation across interconnected components of the liver-brain axis rather than isolated biochemical changes. However, these findings do not establish direct causality between individual steps or a 5-HT3 receptor-dependent mechanism.

Several limitations should be acknowledged. First, 5-HT3 receptor expression, occupancy, and target engagement were not assessed; therefore, the observed effects cannot be attributed specifically to 5-HT3 receptor antagonism. Second, blood ammonia concentrations and glutamine synthetase activity were not measured, and cerebral glutamine should therefore not be interpreted as a direct measure of systemic hyperammonemia or ammonia detoxification. Third, the inflammatory findings were based primarily on semiquantitative immunohistochemistry without complementary quantitative molecular analyses or cell-specific co-localization, limiting conclusions regarding pathway activation and cellular sources. Fourth, standardized neurological or behavioral assessments, EEG recordings, and quantitative measures of cerebral edema were not performed; consequently, the observed biochemical and tissue-level improvements cannot be assumed to represent functional or electrophysiological recovery. Previous TAA studies have demonstrated progressive behavioral and EEG abnormalities associated with HE severity [32,33]. Finally, only female rats were studied, precluding assessment of sex-dependent responses. Future studies incorporating receptor-specific approaches, ammonia-glutamine pathway measurements, quantitative and cell-specific molecular analyses, and functional and neurophysiological assessments in both sexes are required to further define the mechanisms and translational relevance of these findings.

## 4. Materials and Methods

Thirty adult female Sprague-Dawley rats (2–3 months old, weighing 220 ± 20 g) were obtained from the Erzincan Binali Yildirim University Experimental Animal Research and Application Center (Erzincan, Türkiye). Animals were housed under standard laboratory conditions (22 ± 2 °C, 55 ± 10% humidity, and a 12 h light/dark cycle) with free access to standard pellet chow and tap water. After one week of acclimatization, the animals were randomly allocated into five experimental groups (*n* = 6 per group): Control, TAA, OND, TAA+OND (0.5 mg/kg), and TAA+OND (1 mg/kg). All experimental procedures were approved by the Erzincan Binali Yildirim University Local Animal Ethics Committee (30.01.2026 dated, E-85748827-050.04-520561 numbered) and were performed in accordance with the National Institutes of Health Guide for the Care and Use of Laboratory Animals and the ARRIVE guidelines [34]. Biochemical measurements were conducted at the Research and Development Laboratory, Faculty of Medicine, Erzincan Binali Yıldırım University, Erzincan, Türkiye.

TAA-induced acute HE was established using an established experimental protocol of intraperitoneal administration of thioacetamide (TAA, 300 mg/kg/day) for three consecutive days (Days 1–3) [35]. Ondansetron (0.5 or 1 mg/kg, i.p.) was administered once daily for two consecutive days following TAA administration [17]. Control animals received physiological saline according to the same experimental schedule, while the OND group received ondansetron (1 mg/kg, i.p.) without TAA. To minimize dehydration and metabolic disturbances associated with TAA administration, animals received 0.5 mL of 10% dextrose in Lactated Ringer’s solution subcutaneously every 12 h following each TAA injection. Thioacetamide and ondansetron were freshly prepared in sterile saline immediately before administration. On day 6, animals were anesthetized with ketamine (75 mg/kg, i.p.) and xylazine (10 mg/kg, i.p.). Blood, liver, and brain samples were collected for biochemical and histopathological analyses.

### Biochemical Parameters

Liver and brain tissues (50 mg) were homogenized in ice-cold phosphate buffer (50 mM, pH 7.4) and centrifuged at 5000× *g* for 15 min at 4 °C. The resulting supernatants were used for biochemical analyses. Protein concentrations were determined using the Bradford method [36] and all tissue parameters were normalized to protein content.

Catalase (CAT) activity was determined according to Goth [37]. Superoxide dismutase (SOD) activity was measured using the method described by Sun et al. [38]. Myeloperoxidase (MPO) activity was measured according to Bradley et al. [39]. Malondialdehyde (MDA) levels were determined using the method of Ohkawa et al. [40], as modified by Kurt et al. [41]. Nitric oxide (NO) levels were measured using a commercial Nitrate/Nitrite Colorimetric Assay Kit (Cayman Chemical, Ann Arbor, MI, USA) according to the manufacturer’s instructions.

Serum and brain glutamine concentrations were determined using a commercial ELISA kit (Glutamine ELISA Kit, Cat. No. CK-bio-27615, Shanghai Coon Koon Biotech Co., Ltd., Shanghai, China) according to the manufacturer’s protocol.

Brain and liver tissues obtained by necropsy of rats were fixed in 10% neutral formalin solution. The tissues were subjected to routine alcohol-xylene staining procedures and placed on paraffin blocks. Paraffin-embedded tissues were sectioned at 4 µm and stained with hematoxylin and eosin (H&E). Liver sections were evaluated semiquantitatively for hydropic degeneration and hepatocellular necrosis, whereas brain sections were assessed for pyknotic and degenerative neuronal changes. Lesion severity was scored as absent (0), mild (1), moderate (2), or severe (3). Histopathological evaluations were performed by an investigator blinded to the experimental groups.

Brain and liver tissues were stained immunohistochemically. For this purpose, four-micrometer sections were mounted on poly-L-lysine-coated slides, passed through xylene and alcohol series, washed with PBS, and then incubated in 3% H_2_O_2_ for 10 min to block endogenous peroxidase activity. Antigen retrieval was performed in the appropriate retrieval solution using microwave heating at 500 W for two 5 min cycles. Then, the sections were incubated overnight with the primary antibodies in Table 1 at a dilution of 1:200. Subsequently, a Large Volume Detection System: anti-Polyvalent HRP (Thermo Fisher Scientific, Waltham, MA, USA; Cat. No. TP-125-HL) was applied according to the manufacturer’s instructions. After counterstaining with Mayer’s Hematoxylin, the samples were sealed with water-based adhesive and examined under a light microscope. In the examination, immunoreactivity was evaluated semiquantitatively as negative (0), weak (1), moderate (2), strong (3), or very strong (4). The caspase-1 antibody used in this study was not specific for the cleaved/active form; therefore, the analysis represents overall caspase-1 immunoreactivity rather than a specific assessment of active caspase-1.

Statistical analyses were performed using IBM SPSS Statistics (version 20 and 22; IBM Corp., Armonk, NY, USA), and graphs were generated using GraphPad Prism (version 6 and 8; GraphPad Software, San Diego, CA, USA). Data are presented as mean ± SD. Normality was assessed using the Shapiro-Wilk test and homogeneity of variances using Levene’s test. Normally distributed variables with homogeneous variances were analyzed by one-way ANOVA followed by Tukey’s post hoc test. When homogeneity of variance was violated, Welch’s ANOVA followed by the Games-Howell post hoc test was used. Non-normally distributed variables were analyzed using the Kruskal-Wallis test followed by appropriate pairwise comparisons. Histopathological and immunohistochemical scores were analyzed using the Kruskal-Wallis test. A *p* value < 0.05 was considered statistically significant.

Generative artificial intelligence (GenAI) tools were used during manuscript preparation to assist with language refinement and improvement of scientific clarity. All AI-assisted content was critically reviewed, verified, and edited by the authors, who take full responsibility for the final content of the manuscript.

## 5. Conclusions

Overall, OND administration was associated with partial attenuation of metabolic, oxidative, inflammatory, and histopathological alterations in the TAA-induced acute HE model. The effects were tissue- and marker-specific and were generally more pronounced at 0.5 mg/kg than at 1 mg/kg, indicating a non-linear dose-related pattern within the tested dose range. These findings support further investigation of OND in experimental HE; however, the present study does not establish a direct 5-HT_3_ receptor-dependent mechanism or therapeutic efficacy. Further studies incorporating receptor-specific approaches, pharmacokinetic assessment, ammonia and glutamine synthetase measurements, and functional neurological outcomes are required to clarify the mechanistic and translational relevance of these findings.

## Figures and Tables

**Table 1:** Primary antibodies used in immunohistochemical staining of tissues.

Tissue	Primary Antibody	Manufacturer	Catalog No
Brain and liver	IL-1β	Santa cruz Biotechnology (Dallas, TX, USA)	sc-52012
	Caspase 1	BT-Lab (Shanghai, China)	BT-AP01191
	NLRP3	Affinity Biosciences (Changzhou, China)	BF-8029
	NFkB	Elabscience (Wuhan, China)	E-AB-22016
	TLR2	Affinity Biosciences (Changzhou, China)	DF7002
Brain	GFAP	BT-Lab (Shanghai, China)	BT-AP03549

## Data Availability

The original contributions presented in this study are included in the article. Further inquiries can be directed to the corresponding author.

## Abbreviations

The following abbreviations are used in this manuscript:

HE	Hepatic encephalopathy
OND	Ondansetron
TAA	Thioacetamide
GLN	Glutamine
SOD	Superoxide dismutase
CAT	Catalase
MDA	Malondialdehyde
NO	Nitric oxide
MPO	Myeloperoxidase
TLR	Toll-like receptors
NF-κB	Nuclear factor-kappa B
NLRP3	Nucleotide-binding oligomerization domain-like receptor protein 3
IL-1β	Interleukin-1 beta
5-HT_3_	5-hydroxytryptamine type 3
